# Have Historical Climate Changes Affected Gentoo Penguin (*Pygoscelis papua*) Populations in Antarctica?

**DOI:** 10.1371/journal.pone.0095375

**Published:** 2014-04-23

**Authors:** Fabiola Peña M., Elie Poulin, Gisele P. M. Dantas, Daniel González-Acuña, Maria Virginia Petry, Juliana A. Vianna

**Affiliations:** 1 Departamento de Ciencias Ecológicas, Universidad de Chile, Santiago, Metropolitan Region, Chile; 2 Pós-Graduação em Zoologia de Vertebrados, Pontificia Universidade Catolica de Minas Gerais, Belo Horizonte, Minas Gerais, Brazil; 3 Departamento de Ciencias Pecuarias, Universidad de Concepción, Chillán, Chile; 4 Laboratório de Ornitologia e Animais Marinhos, Universidade do Vale do Rio dos Sinos, São Leopoldo, Rio Grande do Sul, Brazil; 5 Departamento de Ecosistemas y Medio Ambiente, Pontificia Universidad Católica de Chile, Santiago, Metropolitan Region, Chile; Estonian Biocentre and Tartu University, Estonia

## Abstract

The West Antarctic Peninsula (WAP) has been suffering an increase in its atmospheric temperature during the last 50 years, mainly associated with global warming. This increment of temperature trend associated with changes in sea-ice dynamics has an impact on organisms, affecting their phenology, physiology and distribution range. For instance, rapid demographic changes in *Pygoscelis* penguins have been reported over the last 50 years in WAP, resulting in population expansion of sub-Antarctic Gentoo penguin (*P. papua*) and retreat of Antarctic Adelie penguin (*P. adeliae*). Current global warming has been mainly associated with human activities; however these climate trends are framed in a historical context of climate changes, particularly during the Pleistocene, characterized by an alternation between glacial and interglacial periods. During the last maximal glacial (LGM∼21,000 BP) the ice sheet cover reached its maximum extension on the West Antarctic Peninsula (WAP), causing local extinction of Antarctic taxa, migration to lower latitudes and/or survival in glacial refugia. We studied the HRVI of mtDNA and the nuclear intron β*fibint7* of 150 individuals of the WAP to understand the demographic history and population structure of *P. papua*. We found high genetic diversity, reduced population genetic structure and a signature of population expansion estimated around 13,000 BP, much before the first paleocolony fossil records (∼1,100 BP). Our results suggest that the species may have survived in peri-Antarctic refugia such as South Georgia and North Sandwich islands and recolonized the Antarctic Peninsula and South Shetland Islands after the ice sheet retreat.

## Introduction

The West Antarctic Peninsula has been described as one of the areas most affected by global warming due to atmospheric temperature increment; it has experimented an increase of 5–6°C during the last 50 years [1]–[4]. The ecosystems of the WAP are principally regulated by ice dynamics, including its extension, periodicity and inter-annual variation [1]. Thus it is expected that changes in atmospheric and ocean temperatures will have a great impact on biological systems, affecting their phenology, physiology and distribution range [5], [6]. At present there is strong evidence related to the effect of climate change on biological interactions and food webs [7]. Also, evidence of differential response between ice-obligated and non-ice-obligated marine mammals and birds has been found [5], [8]–[10].

Penguins have been described as climate indicator species; changes in their populations over the last 50 years have been documented, mainly associated with changes in ice dynamics [11]–[15]. Among the *Pygoscelis* species, *P. adeliae* (Adélie) is the most ice-dependent species; its range reaches higher latitudes and has a circumpolar distribution. In contrast, *P. antarcticus* (Chinstrap) is found almost exclusively around the Antarctic Peninsula [16], while *P. papua* presents a distribution range including most of sub-Antarctic islands and the Antarctic Peninsula. *Pygoscelis papua* (Gentoo) populations have been increasing along the Antarctic Peninsula in the last 50 years; this has been mainly associated with an abrupt increment of temperatures in the Antarctic Peninsula that affects sea ice extension, which modulates access to breeding sites and the establishment of krill stocks. These changes in sea-ice dynamics have allowed southward colonization of *P. papua* populations [12], [13], [17]–[19]. Contrarily, populations of *P. adeliae* and *P. antarcticus* have been decreasing in the WAP, mainly related to the availability of krill [18], [20].

Current global warming has been described by the Intergovernmental Panel on Climate Change (IPCC) without precedents over decades to millennia mainly associated with human activity. Nevertheless, this global trend is framed in a context of historical climate changes, particularly during the Pleistocene, characterized by an alternation between glacial and interglacial periods [21]. These warming and cooling events had an impact on the extension and collapse of ice masses in Antarctic, periodically altering land, fresh water and marine habitats [22]. In the last glacial maximum (LGM∼21,000 BP), Antarctic Peninsula ice sheet reached its maximum extension on the West Antarctic Peninsula (WAP). During the LGM decreasing temperatures and habitat loss were the main limitations for the permanence of biological communities [22]. The thickness of the ice sheet on the WAP, around 1.5–2.5 Km as well as ice shelf extension [23] caused a significant reduction of flora and fauna through local extinction of Antarctic taxa [24]–[26], migration to lower latitudes [27] and/or survival in glacial refugia [28]. There is also evidence that during past climate change that affected ice extension, penguin populations responded by abandoning their breeding colonies towards more profitable habitats [29], [30]. For example, as shown by paleoecological records, Antarctic *P. adeliae* penguins responded by abandoning nest colonies and dispersing to suitable habitats. Richie et al. [31] found the existence of two mitochondrial DNA lineages associated with different refuge histories during the LGM mainly attributed to the expansion of sea ice and loss of ice-free coastal areas required for nesting [29], [30]. In contrast to Antarctic species such as *P. adeliae*, ice-independent species are expected to have been even more affected during the LGM, reducing their distribution to lower latitudes such as sub-Antarctic islands or to refuges near the Antarctic polar front. This has been supported by molecular and fossil evidence that suggests postglacial re-colonization of the Antarctic Peninsula by highly dispersive species such as marine mammals and birds [26], [32], as well as shallow marine invertebrates [33]. For example, molecular studies of *Mirounga leonina* show a post-LGM re-colonization of Victoria Land about 7,500–8,000 BP from Macquarie Island [32]. However, Emslie et al. [34] found that *P. papua* occupation of the Antarctic Peninsula did not occur until the late Holocene; the oldest record for this species is about ∼1,100 BP. Thus, we expected to find a clear recent signal of expansion for *P. papua* of less than 2,000 years. However, if the fossil found by Emslie et al. [34] was an outcome of the re-colonization after the LGM due to the expansion of one or more *P. papua* populations that were isolated during the Pleistocene climate change, we would expect to find an older signature of expansion and a marked genetic structure in the WAP region.

In this study we used mitochondrial DNA (mtDNA) HVR-I and nuclear DNA (nDNA) β*fibint7* sequences to evaluate past changes in the demographic history and population genetic structure of *P. papua*, to assess the dynamics of Antarctic populations during the LGM. Understanding how penguin populations have responded to past climate changes would allow predicting how future changes may affect their population dynamics.

## Methodology

### Sample Collection and DNA Extraction

For penguins capture and handling, we used procedures with the least amount of stress. In order to minimize the disturbances we might be causing in the colonies, the penguins were captured several meters from the nesting sites when the animals left to the water. The penguins were captured using a hand-held net, and then immobilized manually as described by Wilson [35]. During the complete procedure the penguin remains with his head covered to reduce stress and the beak immobilized. Blood samples were collected from adult individuals from each studied colony. Samples were taken with 23 G needles from the brachial or external metatarsal vein (∼0.5 mL) and stored in 96% ethanol for posterior analysis. After samples were collected, the individuals were released in the same place they were captured and they were monitored until finds a shelter or returns to the water.

**Figure 1:**
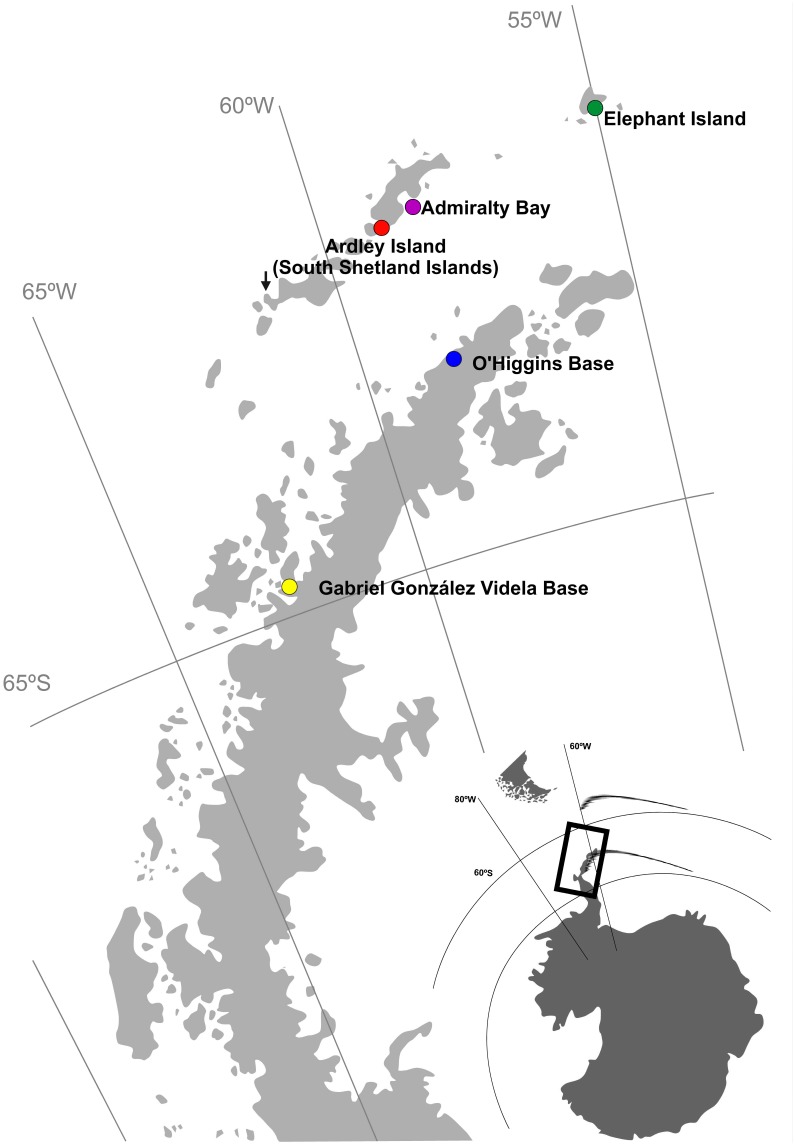
Location of the five studied locations of *P. papua* along the West Antarctic Peninsula. The black arrow indicates the *P. papua* fossil record from Emslie *et al*. (2011).

*Pygoscelis papua* blood samples were collected from five nesting areas in the Antarctic: Elephant Island (61°13′S; 55°21′W); Admiralty Bay (62°23′S, 58°67′W) and Ardley Island (62°13′S, 58°54′W) on King George Island in the South Shetland Islands; and the Chilean Antarctic bases Gabriel González Videla at Paradise Bay (64°49′S, 62°52′W) and Libertador Bernardo O’Higgins at Covadonga Harbor (63°19′S, 57°54′W; Figure 1) on the West Antarctic Peninsula. All samples were collected with permits in accordance to Annex II, Article 3 of the Protocol on Environmental Protection to the Antarctic Treaty, and the regulation from the Scientific Committee on Antarctic Research (SCAR) provided by the Chilean Antarctic Authorities (permits INACH 44/2012), and the Brazilian Antarctic Authorities through the PROANTAR and Environmental Ministry (N°21/2011 and N°20/2012). The INACH and PROANTAR permit included authorization for sample collection for all locations and to develop research on Ardley Island and Admiralty Bay, the only locations for which a specific permit was required. Bioethics permit was provided by Universidad de Concepción and Pontificia Universidad Católica de Chile which was required to obtain INACH permits.

### DNA Amplification and Sequencing

Genomic DNA was extracted from blood samples employing saline method [36]. Hypervariable Region I of mtDNA (HVR-I) was amplified employing primers from Roeder et al. [37]: tRNAGlu (5′-CCCGCTTGGCTTYTCTCCAAGGTC-3′) and AH530 (5′- CTGATTTCACGTGAGGAGACCG-3′). Each 25 µl PCR reaction contained 0.4 µm each primer, 200 µm each dNTP, 1.5 mM MgCl_2_, 1 µl 10X PCR reaction buffer and 1 U Taq Polymerase Invitrogen. Thermocycler conditions were an initial denaturing at 95°C for 5 min, followed by 38 cycles of 95°C for 30 s, 55°C for 45 s and 72°C for 15 min, and a final extension at 72°C for 7 min.

Intron 7 of the β-fibrinogen gene (β*fibint7*) was amplified using the primers Fib7U (5′-CAGGACAATGACAATTCAC-3′) and Fib7L (5′-GTAGTATCTGCCATTAGG-3′) [38]. The PCR reaction mixture contained 0.4 µm each primer, 200 µm each dNTP, 1.5 mM MgCl_2_, 1 µl 10X PCR reaction buffer and 1 U Taq Polymerase Invitrogen. Thermal conditions were an initial denaturing at 95°C for 10 min, followed by touchdown of 10 cycles of 95°C for 15 s, 60–50°C for 30 s and 72°C for 45 s, a second stage of 35 cycles of 95°C for 15 s, 50°C for 30 s and 72°C for 45 s and a final extension phase of 72°C for 30 min. All PCR products were visualized in 2% agarose gels and sequenced using the service of Macrogen, South Korea. Posterior analysis and editing of sequences employed Proseq v 2.0 [39] obtaining 371 bp for HVR-I and 1014 bp for βfibint7. Sequences were deposited in GenBank under accession numbers KF717669–KF717743 for mtDNA HVR-I, and KF803989–KF803995 for nDNA βfibint7.

### Data Analysis

The nuclear marker βfibint7 haplotype reconstruction was developed employing the Phase module in DnaSP v. 5.0 [40]. Genotype reconstruction was based on the algorithms of PHASE v.2.1 [41], [42], using coalescent Bayesian methodology to infer haplotypes. Standard diversity indexes (K, S, Hd, π and ∏) were calculated for both HVR-I and βfibint7 using DnaSP v. 5.0 [40]. Genealogical relationships between haplotypes were established employing the median joining algorithm implemented in Network v. 4.611 [43]. Assignment weights for frequent mutation steps (>6 times) were changed from 10 to 5 to eliminate loops and simplify the tree.

To evaluate population genetic structure we estimated *F_st_* and *Φ_st_*, using Arlequín v.3.5.1.3 [44]. Phylogeographic structure was evaluated employing Permut [45], [46], considering 10,000 permutations. Isolation by distance was evaluated with the Mantel test (10,000 permutations) implemented in Arlequín v.3.5.1.3 [44]. Spatial structure was assessed using Geneland 4.0.2 [47], [48] occupying a correlated analysis and 1,000,000 iterations.

Mutation-drift equilibrium was tested employing Tajima’s *D* and Fu’s *Fs* indexes [49], [50] in Arlequín v.3.5.1.3 [44]. Due to the low genetic diversity present in βfibint7, demographic inference analyses were not performed on this marker to avoid overestimation. Mismatch distribution was reconstructed to evaluate past population changes, estimating both demographic and expansion parameters under the instantaneous growth model [51]. Expansion time was estimated from 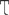 = 2 µt, µ: mutation rate substitution/site/year employing a 55% mutation rate for HVR-I [52]. Effective population size and population demographic history of penguin populations were estimated using Bayesian inference employing BEAST v.1.7.4 [53]. The evolution model for the sequence dataset was selected employing JModelTest under Bayesian inference criteria (BIC) [54]. For HVR-I, the HKY+I+G model was selected for the data set. Selection of the inference method was performed using Bayes Factor [55], [56], which selected the Bayesian skyline plot for *P. papua* with a relaxed molecular evolution clock and exponential distribution using 0.55 s/s/ma mutation rate. Graphic representation was developed in Tracer v 1.5 [57].

## Results

We identified a total of 75 haplotypes (n = 150) for HVR-I of *P. papua* (371 bp), with high levels of overall haplotype diversity (h = 0.982), ranging from 0.924 to 0.975 between localities, and a reduced level of nucleotide diversity (π = 0.01288), ranging from 0.01009 to 0.01599. For *βfibint7* (1014 bp), a total of 5 haplotypes (n = 176) were found, with haplotype diversity of 0.492 and reduced nucleotide diversity (π = 0.00091) as expected for a nuclear marker with low mutation rate (Table 1).

**Table 1:** Genetic diversity index for studied locations of *P. papua*.

	Localities	N	K	S	Hd	π	∏
HVR-I	Total	150	75	72	0.982	0.01288	4.779
	Elephant Island	16	12	26	0.958	0.01599	5.933
	Admiralty Bay	26	17	24	0.966	0.01277	4.738
	Ardley Island	35	17	23	0.955	0.0126	4.676
	O’Higgins Base	40	17	24	0.924	0.01009	3.745
	GGV Base	33	24	31	0.975	0.01211	4.492
βfibint7	Total	176	5	3	0.492	0.00091	0.919
	Ardley Island	80	4	3	0.45	0.00086	0.876
	O’Higgins Base	44	4	3	0.53	0.001	1.016
	GGV Base	52	3	2	0.531	0.0009	0.917

Genetic diversity indexes for 371 bp for mtDNA HVR-I and 1014 bp for βfibint7 of *P. papua,* including the overall and population sample size (N), the number of haplotypes (K), polymorphic sites (S), Haplotype diversity (Hd), nucleotide diversity (π) and average number of difference between sequences (∏).

GGV: Gabriel González Videla Base.

**Figure 2:**
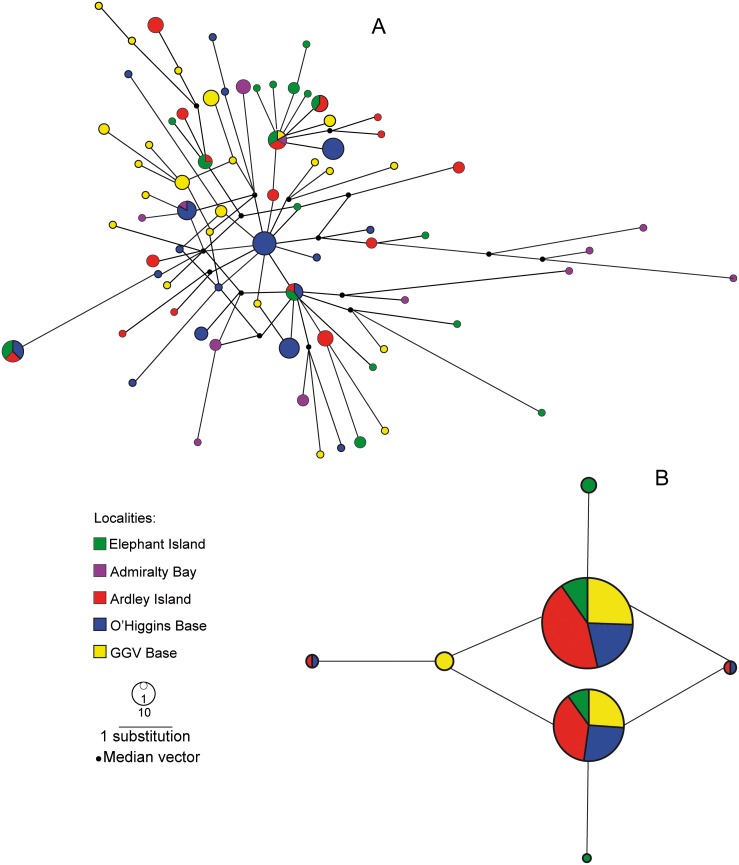
Median Joining Network of mtDNA HVR-I (a) and βfibint7 for *P. papua* (b). GGV: Gabriel González Videla Base.

The median joining network for mtDNA HVR-I reflects the high levels of genetic diversity; there were shared haplotypes between localities. However, only one shared haplotype was found between the southernmost GGV and other localities (Figure 2a). The median joining network for *βfibint7*, even if there were low levels of genetic diversity, shows the existence of shared haplotypes between all localities (Figure 2b).

**Figure 3:**
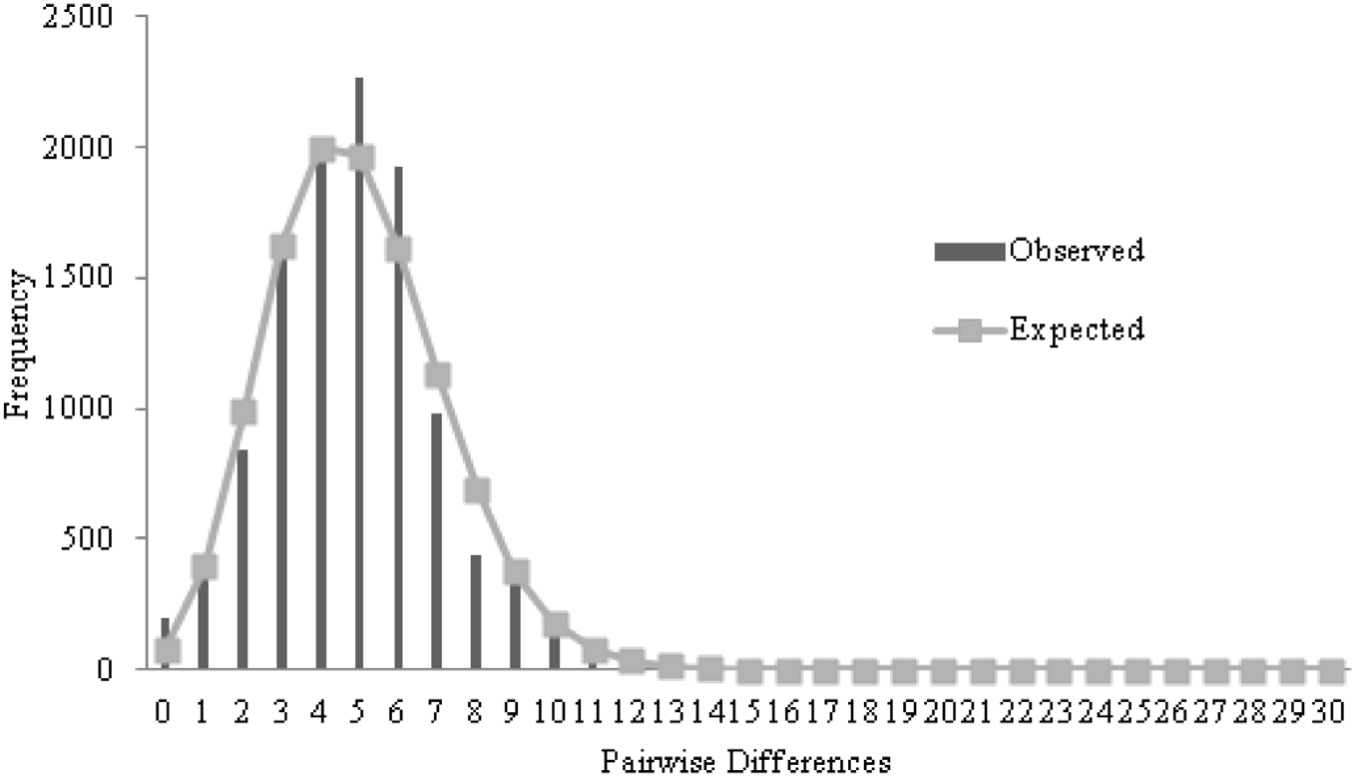
Mismatch Distribution for mtDNA HVR-I for *P. papua*.

**Figure 4:**
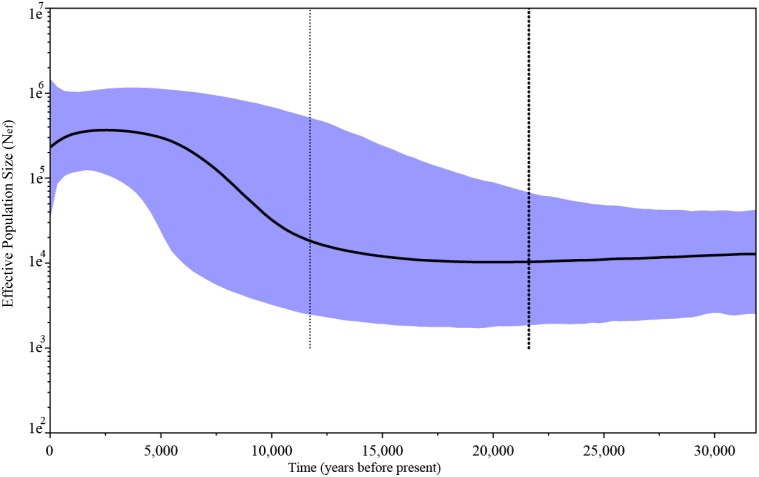
Skyline Plot mtDNA HVR-I for *P. papua*.

Fu’s Fs and Tajima’s *D* test for HVR-I for *P. papua* was not significant (*Fs* = −7.59286, p = 0.0206; *D* = −1.01107, p = 0.1644, respectively). Mismatch distribution (Figure 3) showed a unimodal shape, suggesting a recent expansion (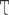 = 4.924), which was estimated at about 12,000 BP. The Bayesian skyline plot for *P. papua* (Figure 4) also suggests a signature of recent expansion around 13,000 BP with the Most Recent Common Ancestor time (MRCA) around 21,500 BP. The increase in effective population size (Ne) for *P. papua* is about one order of magnitude.

Spatial genetic analysis to infer subpopulations (K) indicated an absence of clusters (K = 1). However, a reduced but significant population genetic structure was observed between pairwise populations for HVR-I for *P. papua*, with *F_st_* values ranging from 0.027 to 0.052 and *Φ_st_* ranging from 0.041 to 0.102 (Table 2). The only non-significant values of *F_st_* and *Φ_st_* were found between the Ardley Island and Admiralty Bay sites, located only 30 km apart in King George Island. We did not find a significant *s*ignature of isolation by distance (r =  −0.046770, p = 0.5015).

**Table 2:** Population genetic structure between locations of *P. papua*.

*F_st_*	Elephant Island	Admiralty Bay	Ardley Island	O’Higgins Base	GGV Base
Elephant Island	–		0.01314	−0.00576	−0.00325
Admiralty Bay	0,03298***	–			
Ardley Island	0,04019***	0,017**	–	−0,00158	−0,00359
O’Higgins Base	0,05226***	0,04326***	0,047***	–	−0,01103
GGV Base	0,03094***	0,02691***	0,0283***	0,04458***	–
***Φ_st_***	**Elephant Island**	**Admiralty Bay**	**Ardley Island**	**O’Higgins Base**	**GGV Base**
Elephant Island	–				
Admiralty Bay	0,09572***	–			
Ardley Island	0,06889***	0,1221	–	−0,00135	−0,00765
O’Higgins Base	0,06187***	0,04078**	0,04832***	–	−0,01421
GGV Base	0,09048***	0,1021***	0,08264***	0,0829***	–

*F_st_* and *Φ_st_* values between the five pairwise locations of *P. papua*. Values below the diagonal are mtDNA HVR-I; above the diagonal, βfibint7. **<0.05; ***0.01.

GGV: Gabriel González Videla Base.

## Discussion

Molecular data support the hypothesis that *P. papua* populations were affected by Pleistocene climate changes. This can be evidenced by high levels of genetic diversity, an absence of population expansion signature from Fu’s Fs and Tajima’s *D*, whereas the expansion signature detected by Bayesian skyline plot can be associated to a recolonization scenario (see below). We found a signature of population expansion for *P. papua* after the LGM (12,000–13,000 BP), and the MRCA around 21,500 BP. The date of expansion calculated is coincident with the retreat of the ice sheet cover in the maritime Antarctic (Antarctic Peninsula and adjoining islands, [58]). Nevertheless, fossil evidence suggests that *P. papua* would have established colonies in South Shetland Islands much later, around 1,100 BP during the late Holocene [34]. However fossil records for *P. papua* are very scarce and it is possible that older evidence has not yet been recovered. There is also the possibility that our expansion time could be overestimated due to the great amount of genetic diversity, which can be attributed to a colonization of maritime Antarctic from a large number of migrant individuals from different refuges. Dinechin et al. [59] detected the existence of three different clades in *P. papua*: one associated with sub-Antarctic islands near the Indian Ocean (Kerguelen), another associated with the Falkland Islands and the third associated with maritime Antarctic and peri-Antarctic Islands (i.e. South Georgia, Gough, Sandwich Islands [58]). The existence of a unique linage in the maritime Antarctic suggests that during the LGM more than one colony remained near the Antarctic Peninsula, in the East Antarctic Peninsula and some maritime and peri-Antarctic island refugia, as has been proposed for another Antarctic species [28]. Contrary to the expected for this scenario a severe signature of bottleneck was not detected, and all populations exhibited high levels of genetic diversity. These results may indicate that the colonization of deglaciated areas involved a large number of individuals from multiple locations or refuges, rather than through successive founder effects as has been proposed for many Antarctic species [33], [60]. Therefore, our results suggest that the species may have survived in peri-Antarctic refugia such as South Georgia and North Sandwich Islands and recolonized the Antarctic Peninsula and South Shetland Islands after the ice sheet retreat. Recent colonization from multiple sources is also supported by the low levels of genetic structure found in our sampling area as well as the absence of isolation by distance among sites.

Although low, mtDNA HVR-I genetic differentiation among localities was significant between most of the pairwise comparisons, except between the two closest localities in King George Island (Admiralty Bay and Ardley Island); it was not possible to detect any genetic structure with nDNA βfibint7. However, this discrepancy is likely related to the difference in substitution rates and diversity between the markers [61], [62]. Alternatively, differences in genetic structure between nuclear (biparental markers) and mitochondrial markers (maternal lineage) can result of differential migration or dispersal rates between males and females. Philopatric behavior has been well established in pygoscelid penguins; adults breed in or near their natal colony [63]. For instance, *P. papua* displays a relatively high degree of site fidelity, between 89–100% in South Georgia [64] and 60% in King George Island [65]. Unfortunately, no information is available about differences in philopatry between sexes in this species. It is thus not possible to support a female-biased philopatry hypothesis to explain the discrepancy between mitochondrial and nuclear markers. Moreover, in the closely related *P. adeliae,* [66] found that nest site fidelity is higher for males (98.9%) than for females (65.3%). Another study found also that the majority of males (62%) returned to the same nest during the 4 years of study, while only 29% females returned to the same nest [67]. Although philopatric behavior is not concordant with a low genetic structure found for mtDNA, which is a maternal lineage molecular marker, the colonizing ability of *P. papua* could be an explanation. Gentoo penguins are apparently excellent colonizers of new breeding territory, especially at their southern range boundary [68]. Moreover, range expansion erases phylogeographic structure, as suggested for *P. adeliae* [31]; this pattern was observed for other Antarctic bird species. The south polar skua has undergone demographic and/or spatial expansions and is considerably less phylogeographically structured than the brown skua [69]. Additionally, it has been proposed that philopatric conduct may be related to stable environmental conditions [70]. Moreover, Dugger et al. [71] found that environmental perturbations can increment dispersal rate among Adélie penguins; they reported that in periods of high environmental adversity penguins tend to increment their movement rates in all colonies and in all directions. Consistent with this idea of high dispersion in the face of climate constrains, Korczak-Abshire et al. [72] studied AFLP for *P. antarcticus* in two localities from King George Island, finding no or only weak population genetic structure. Additionally, Roeder et al. [73] using microsatellites also found reduction in and even absence of genetic diversity for *P. adeliae* around the Antarctic. Considering this information, it is expected that low values of genetic structure may be associated with a relaxing of philopatric behavior in *P. papua*, allowing movement and establishment of colonies of individuals from different source colonies.Further studies along the entire *P. papua* distribution using multilocus markers will help to completely understand the evolutionary history of this species, and the results of biparental variable molecular markers (e.g. microsatellite loci) can be compared with our results of population genetic structure from mtDNA (maternal lineage) to further understand the species behavior.

## Conclusions

Understanding how past climate change affected species demography could help to elucidate the impact of current global warming and to predict future trends in species. The study of *P. papua* demography allowed us to detect that in the past *P. papua* maintained a large genetic diversity associated with large effective population size. Although it has been described as a sub-Antarctic species, our results suggest that the Gentoo penguin probably persisted in peri-Antarctic refugia during the LGM, being able to (re)colonize the maritime Antarctic during warmer periods (such “penguin optimum” period, Baroni and Orombello, 1994). According to this, *P. papua* may be considered as a resilient species which may have survived the extreme cold temperature during the LGM, and is obtaining benefits from the recent climate change in the maritime Antarctic. Over the last 30 years Gentoo penguin populations have been increasing in the WAP [18], [74], colonizing and establishing new colonies with fast growth rates along the southern boundary [20], and it is rapidly adapting to the shifts in the marine food web due to its opportunistic foraging [68]. It is expected that in the future *P. papua* will be a dominant species, at least in the Antarctic Peninsula, as temperatures increase in the Antarctic.
